# Spectral Analysis of Torsional Vibrations Measured by Optical Sensors, as a Method for Diagnosing Injector Nozzle Coking in Marine Diesel Engines

**DOI:** 10.3390/s21030775

**Published:** 2021-01-24

**Authors:** Sebastian Drewing, Kazimierz Witkowski

**Affiliations:** Faculty of Marine Engineering, Gdynia Maritime University, 81-225 Gdynia, Poland; k.witkowski@wm.umg.edu.pl

**Keywords:** marine propulsion, marine power plants, condition monitoring, torsional vibration spectra, diagnostics, marine diesel engines, coked injector, frequency, harmonic orders

## Abstract

The study aimed to verify whether it is possible to diagnose the coking of a marine diesel engine injector nozzle by performing a spectral analysis of the crankshaft’s torsional vibrations. The measurements were taken using laser heads, clocked at 16 MHz. The reasons for selecting this type of optical sensors are described as well. The tests were carried out under laboratory conditions, using a test stand with a Sulzer 3AL 25/3 engine, operating under a load created by a Domel GD8 500–50/3 electric generator. A unique method is presented in the paper, which enables the measuring and calculation of torsional vibrations of engine crankshafts. The method was developed at the Chair of Marine Power Plants at the Maritime University of Gdynia. It has been proven that the distribution of differences in the values of individual harmonic components depends on the location of a defective injector nozzle in the cylinder.

## 1. Introduction

Internal combustion engines with reciprocating pistons are one of the most complex pieces of machinery operating in ship engine rooms. In the course of their operation, frequent defects affecting the fuel system, including injector nozzles, are experienced. The reasons for this are the long-term supply of the engine with fuels of poor quality or with high contents of bio-additives, and poor technical condition of the system piston, piston rings and cylinders, resulting in the combustion of engine oil. The frequency of incidents of this type has been increasing since 2012, mainly due to the introduction of low-sulfur fuels used that are used in the marine industry to ensure compliance with ISO 8217:2012 and ISO 8217:2017 standards mandated by the International Maritime Organization. Low sulfur fuel oils (LSFOs) are characterized by a higher catalytic mud (consisting, inter alia, of very hard aluminum and silicon compounds) content (up to 60 ppm), compared to fuels with a high sulfur content. This is caused by the fact that fine particles end up in low-sulfur by-products of the refining process which are then mixed with residual fuels to reduce sulfur content. Marine engine manufacturers, such as MAN and Wärtsilä, recommend a maximum content of these hard fractions of 15 ppm [1].

The current methods used for diagnosing the operation of fuel systems of internal combustion engines are mainly based on indirect parametric methods that rely on the analysis of variability in selected parameters, induced by damage to the injection system. On the other hand, methods based on injection system pressure measurements seem to be much more effective [2]. The main disadvantage of this solution is the fact that it cannot be usually applied in engine rooms of sea-going vessels and ships operating on inland waters. This is caused, inter alia, by the high cost of piezoelectric pressure sensors, the requirements of classification societies that prohibit any welded and soldered joints, and the need to use certified covers on the pipelines concerned [3,4]. Research performed over the years, as well as experience gathered while evaluating practical applications, continuously increases the degree of efficiency and diagnostic suitability of information concerning machine vibrations. The operational diagnostics of ship propulsion shafts, based on the measurements of mechanical vibrations, consists of measuring specific physical quantities that characterize vibrations of selected driveline components [5]. Such quantities include the displacement, velocity, and acceleration of vibrations. However, diagnosing machinery and mechanisms aboard a vessel, using vibroacoustic methods, creates a number of problems. This stems primarily from a significant concentration of machinery and equipment in a confined area. Other factors include common power sources, the connection of machine foundations by fixed elements of the hull, and, finally, the fact that individual components operate, simultaneously, inside and outside the hull. This results in the overlapping of vibrations from different sources [6,7,8,9]. Torsional vibrations of drive shafts, which are least susceptible to such phenomena, seem to be the most difficult to measure. The crankshaft of an internal combustion engine is a flexible component that is exposed to periodic forces generated by gases and mass. These forces serve as impulses that generate different forms of forced vibrations of the shaft. In a piston engine, these forces generate—in addition to bending and longitudinal oscillations—torsional vibrations as well [10]. A defect of the injector nozzle of an internal combustion engine injector reduces the gaseous forces in a given cylinder, thus changing the distribution of torque affecting the crankshaft.

The search for a reliable method allowing the recreation of the pressure values developing inside a cylinder, based on indirect measurements, has been ongoing for over 30 years. The focus is two-fold: on the one hand, measurements of instantaneous angular velocity of the crankshaft are performed [11,12,13,14], while on the other, measurements of the engine’s lateral vibrations [15,16,17,18] are taken.

These studies prove that both lateral vibrations and angular velocity contain information about the pressure inside engine cylinders but pertain to different frequency ranges. For the reasons described above, i.e., due to the fact that vibrations overlap, the decision was made to rely, in our study, on instantaneous angular velocity measurements.

Rotational speed fluctuations are mainly caused by low-frequency portions of the pressure curve and, therefore, angular velocity is much less sensitive to sudden pressure changes, compared to lateral engine vibrations.

There are some commonly known ways to diagnose engine operation based on the fluctuations of angular velocity of the flywheel. Under specific engine operating conditions, changes in instantaneous angular velocity may be a source of information on the incorrect operation of specific cylinders [19,20,21,22]. Considering the fact that instantaneous velocity is a derivative of displacement in time (displacement is the function of time, thus it is a classic example of a derivative function based on its argument), a decision was made to check whether this signal may be relied upon for diagnostic purposes.

In consideration of the planned future application of the experiment’s results in diagnosing interference/damage of medium- and high-speed marine diesel engines in actual engine room conditions, the selection of an appropriate measurement method was crucial. Vibrations present in a vessel’s engine room are caused both by operating machinery and auxiliary devices, as well as by the ship’s hull that is exposed to waves, precluded the use of most contact-based (i.e., rotating together with the shaft) and contactless torsional vibration measurement methods.

High cost and difficulties experienced while ensuring rigid installation of the measuring head (making sure that it does not move in relation to the rotating shaft), preclude the use of the contactless method based on laser interferometers. Unfortunately, the use of cheap and popular passive magneto-resistive or magneto-inductive sensors was ruled out as well—as was the use of induction sensors relying on the Hall effect, because they generate an insufficient number of pulses per revolution, need to be positioned very close to the shaft (less than ~5 mm), and are sensitive to lateral vibrations [23].

Contact-based methods relying on the use of relatively cheap and commonly applied piezoelectric vibration acceleration transducers could not be used either. Installation of accelerometers poses a risk of the instrumentation being disconnected due to excessive centrifugal loads. The centrifugal force affecting a typical AT3/500 piezoelectric accelerometer that weighs 90 g and is mounted on a shaft with the diameter of 0.2 m equals 222 N at 1500 rpm (described method for diagnosing injector nozzle coking is also planned for low power generators). Additionally, an expensive telemetric system or “sensitive” slip rings are required to transmit the acceleration signals obtained. Acceleration values are measured. The angle of torsion is obtained by integration. The absolute reference position is not available, and thus processing in the domain of angles is impossible as well [24].

For reasons similar to those applying to accelerometers, another contactless method based on tensometric bridges was excluded as well. A method using the newest sensors with fiber grating (FBG—fiber Bragg grating) could not be applied either [25].

Having excluded the above-mentioned methods, a contactless method relying on the use of optical sensors was the only option left. There are many types of optical sensors available on the market, but the majority of them are designed to detect objects. In order to measure angular velocity, encoders which use two types of tapes are most commonly used, depending on whether they are to be glued around the shaft (with zebra tape) or attached to the shaft (disc/zebra disc). Zebra disks and tapes are available in a variety of strip widths in order to adjust the number of pulses per revolution to the diameter of the shaft.

This method was rejected as well due to an insufficient number of pulses per revolution. A decision was made to choose a contactless method that relies on incremental encoders, taking into account the following:

(a) Measurement accuracy resulting from a large number of pulses per revolution;
(b) Absolute reference to the accurate identification of the phase and processing in the domain of angles;
(c) Ability to mount on the free ends of the shaft of a generator set.

Manufactured for industrial applications by the leading brand of Leine Linde, incremental encoders generate up to 10,000 pulses per revolution. Attempts were made to use the encoders of this particular manufacturer for taking the measurements. Unfortunately, due to the length of the free end of the vessel’s engine shaft and, consequently, the rather considerable transverse vibration amplitude values, frequent ruptures of the encoder’s nylon driving shafts were experienced. Enamor Sp. z o.o., a Polish manufacturer of electronic ship optimization and monitoring gear, offered similarly priced upgraded ETNP-10 encoders with laser heads clocked at 16 MHz. Those heads were used (based on our proprietary method) to determine displacement changes of two shaft ends (i.e., to measure torsional vibrations) [26,27].

## 2. Materials and Methods

**Figure 1:**
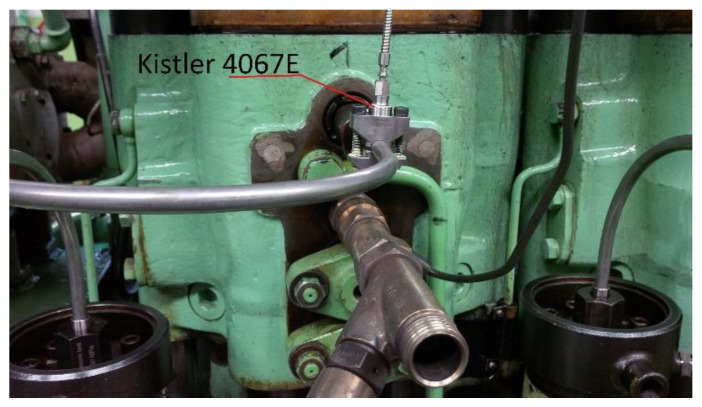
Installation of the Kistler 4067E injection pressure sensor (in the background, the electric cable of the Kistler 6353A24 combustion pressure sensor protruding from the indicator cock).

The measurements were taken on a laboratory test-stand at the Chair of Ship Power Plants of the Maritime University of Gdynia, equipped with a diesel–electric unit (DEU) operating at the speed of 750 rpm. The unit consisted of a three-cylinder Sulzer 3AL25/30 diesel piston engine and a Domel GD8 500-50/3 three-phase synchronous generator. The electricity generated is released by the generator to a water blade resistor. The engine is of the supercharged variety and is equipped with a VTR 160 Brown-Boveri turbocharger with an intercooler. The characteristics of the generator are presented in Table 1. An electronic, stationary Unitest 2008 indicator was used to measure and record pressure waveforms. The measuring system included a recorder with a power supply, three Kistler 6353A24 piezoresistive combustion pressure sensors (reads the pressure with an error < ±0.75, operating range from 0 to 20 MPa), three Kistler 4067E piezoresistive injection pressure sensors (reads the pressure with an error smaller than ±0.8, operating range from 0 to 300 MPa) (Figure 1), and an angular position decoder with an integrated sensor operating with the resolution of 720 pulses per crankshaft revolution.

The recorder communicated with a PC via a USB 2.0 interface. The indicator recorded combustion and fuel injection pressure every 0.5° of crankshaft rotation, based on sixteen full engine cycles, i.e., 32 crankshaft rotations, rendering 1440 pressure measurements per one engine cycle (720 degrees of crankshaft rotation).

A modified ETNP-10 redundant measuring system was used to measure torsional vibrations of the DEU shaft. It consisted of the following:

(a) Two laser heads;
(b) An electronic block, converting the voltage signal from the measuring heads into digital records;
(c) A Saia Burgess Controls programmable logic controller (PLC) for data recording.

**Figure 2:**
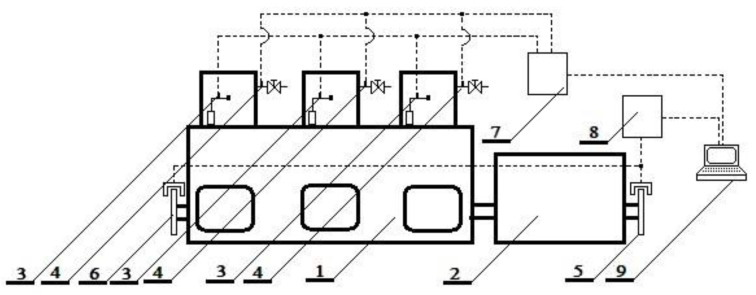
Block diagram of the test stand: 1—SULZER 3AL25/30 marine diesel engine; 2—Domel GD8 500-50/3 synchronous generator; 3—Kistler 4067E sensors for measuring pressure in the injection system; 4—Kistler 6353A24 sensors for measuring combustion pressure; 5,6—ETNP-10 laser heads tracking the movement of the perforated disc; 7—Unitest 2008 indicator; 8—ETNP-10 measuring and control block; 9—computer system for recording measurement data.

**Figure 3:**
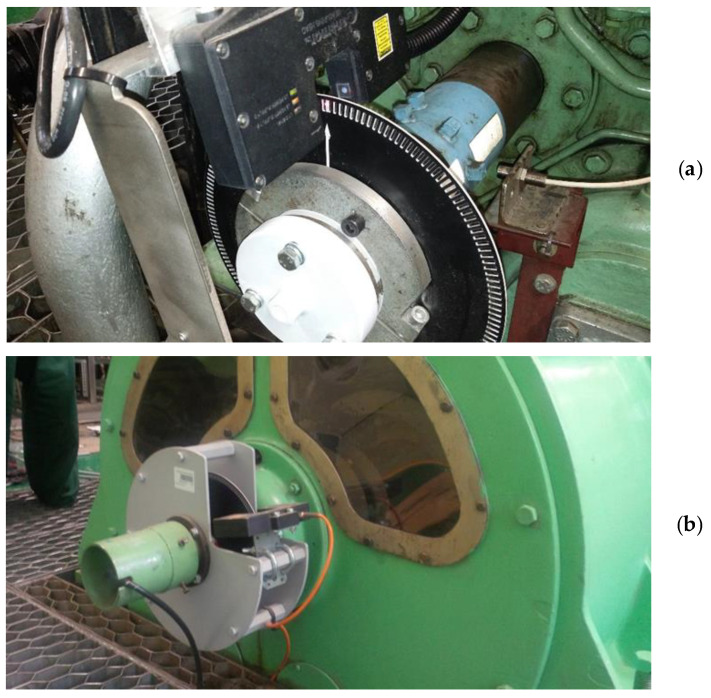
ETNP-10 laser heads are mounted on both free ends of the shaft and track the movement of perforated discs with 180 symmetrical slots along their perimeter ((**a**)—view from the engine side, (**b**)—view from the generator side)).

The signal was processed and recorded in the measuring and control block (Figure 2). The laser heads, being the source of the shaft torsion signal, tracked the movement of two perforated discs with 180 symmetrical slots along their perimeter (Figure 3).

The diesel–electric unit (DEU), operating in the capacity of a vibration signal generator, is a very complex system. This results partly from the following:

(a) As the load of the generator changes, a phase shift occurs between its electromotive force and the voltage in the mains to which electric energy is generated. Any further increase in the load on the unit elevates the value of the phase shift, which also affects the distribution of torsional moments of the drive shaft;
(b) In order to achieve the run uniformity factor of ≤1/250, the unit was equipped with a heavy flywheel. The rotor of the generator is heavy as well;
(c) The shaft is of the resilient variety.

The laser head emitted a laser beam with a frequency of 16 MHz, which was directed onto a photodiode. Assuming sensitivity of photodiode at level ∓10 impulses, the accuracy of measurement depends on the engine’s revolutionary speed, and medium speed diesel engines typical for marine electro-generators reach the level of 0.015% [28]. The gaps and teeth, when passing through the light beam, create groups of signals in the form of a number of pulses with the value of “1” as the light passes through a gap, and of “0” when the light is covered by a tooth. The measured values must be related to angular positions of the shaft. For this purpose, an additional gap was created in the tooth of the first disc. The disc was positioned in such a way that the additional gap corresponded to the top dead-center of the first cylinder (Figure 3a). In addition, the gap serves as a signal that triggers the measurement. The electronic system recognizes two types of signals, so both the gap and the tooth provide information about the instantaneous angular velocity of one of the discs. Then, in order to calculate the torsion of the DEU shaft, the method developed at the Chair of Ship Power Plants was applied, consisting of:

(a) Counting the pulses (*i*_1*i*_) generated by the first measuring head while the first perforated disc moved by two teeth and two gaps (i.e., by 4 degrees of the crankshaft’s rotation). Because the validation of results is based on indicator charts, a gradual measure of the angle was adopted;
(b) Counting the pulses (*i*_2*i*_) generated by the second measuring head while the second perforated disc moved by two teeth and two gaps (i.e., by 4 degrees of the crankshaft’s rotation);
(c) Calculating the time (*t*_1*i*_) in which two teeth and two gaps of the first disc moved by 4 degrees of the crankshaft’s revolution:
  (1) $$t_{1i}=\frac{i_{1i}}{f}$$
  where *f* is the frequency of the laser beam emitted by the measuring head (16,000,000 Hz);
(d) Calculating the time (*t*_2*i*_) in which two teeth and two gaps of the second disc moved by 4 degrees of the crankshaft’s revolution:
  (2) $$t_{2i}=\frac{i_{2i}}{f}$$
(e) Calculating the mean angular velocity (*ω*_1*i*_) for the movement of the first disc by 4° of the crankshaft’s revolution:
  (3) $$\omega_{1i}=\frac{4^{\circ }}{t_{1i}}$$
(f) Calculating the mean angular velocity (*ω*_2*i*_) for the movement of the second disc by 4° of the crankshaft’s revolution:
  (4) $$\omega_{2i}=\frac{4^{0}}{t_{2i}}$$
(g) Calculating the displacement of the second disc (*φ*_2*i*_), assuming that the displacement of the first disc (*φ*_1*i*_) was increasing every 4° of the crankshaft’s revolution (i.e., it equaled 4; 8; 12; 16°… of the crankshaft’s revolution), meaning that the displacement of the second disc was the product of the second disc’s velocity and the time during which the two teeth and two gaps of the first disc moved by 4° of the crankshaft’s revolution:
  (5) $$\varphi_{2i}=\omega_{2i}\cdot t_{1i}$$
(h) Adding all partial displacements of the second disc in order to obtain the total displacement value of the second disc (*φ*_2_). The system measures displacements by one section, consisting of two teeth and two gaps, which is equal to 4° of the crankshaft’s revolution. This means that one full rotation is divided into 90 sections. The system measures 10 crankshaft revolutions, so the total number of sections equals 900:
  (6) $$\varphi_{2}=\varphi_{2i}+\sum_{i=1}^{900}\varphi_{2i}$$
(i) Adding all partial movements of the first disc (*φ*_1_):
  (7) $$\varphi_{1}=\sum_{i=0}^{900}\varphi_{1i}$$
(j) Calculating torsion fluctuations (*φ*) by subtracting the sum of the first disc’s displacements from the sum of the second disc’s displacements:
  (8) $$\varphi^{o}=\sum_{i=0}^{900}\varphi_{1i}-(\varphi_{2i}+\sum_{i=1}^{900}S_{2i})=\varphi_{1}-\varphi_{2}$$

The data obtained in the course of the experiment were collected at equal time intervals (determined by the frequency of the measuring head). They are periodic and continuous, and can therefore be subjected to spectral analysis [29]. As proven in [30], in the case of lateral vibrations of intermediate and helical shafts, amplitudes of the frequency components, and their changes recorded during engine operation may provide a detailed information about local resonance phenomena. This allows the identification and localization of a defect of a specific element, e.g., a bearing, or to detect excessive misalignment between shaft axes.

In consideration of the above, a decision was made to verify whether it was possible to detect fuel system defects based on the spectral analysis of torsional vibrations in a diesel–electric unit. Due to the limited computing power of the hardware available and due to the fact that digital signal analysis is based on the discrete Fourier transform (DFT), the decision was made to use the fastest version based on the Cooley and Tukey algorithm, known as FFT (fast Fourier transform) [31]. The Hamming window was used as the smoothing window (being a modified version of the Hanning window), because it allows the obtaining of a good amplitude accuracy and frequency resolution [32].

## 3. Results and Discussion

The engine was adjusted statically before the laboratory tests commenced. Standby and prime-rated diesel generator sets are designed to operate between 50 and 85% of the full nameplate, while continuous-rated diesel generator sets are optimized between 70 and 100% maximum continuous rating (MCR). Due to the fact that:

(a) The use of the method is planned for use as an on-line diagnostic system on autonomous and unmanned ships;
(b) Failures introduced on one cylinder increase the load on other defect-free cylinders, which often results in exceeding the alarm thresholds of permissible exhaust gas temperatures, the most universal level 70% of loading of the diesel–electric unit was adopted.

Three main research tasks were performed for the generator power rating of 250 kW, which corresponded to 70% of its MCR.

Task 1. Measurement of pressure in cylinders, pressure in the fuel injection system, and torsional vibrations in the diesel–electric unit’s shaft—performed in a defect-free ship engine.

**Figure 4:**
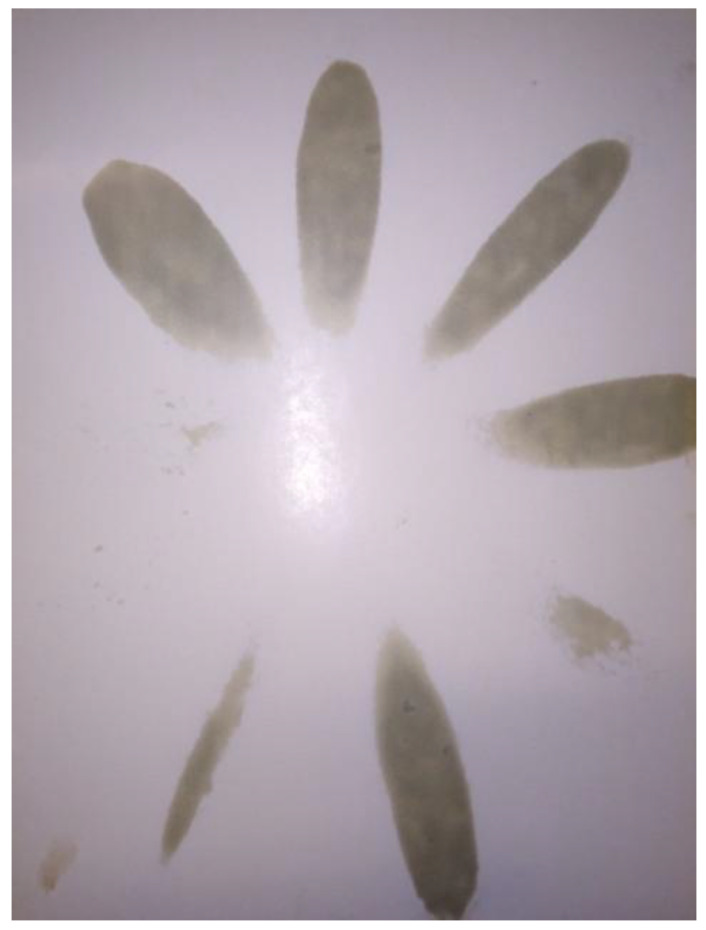
Fuel stream sprayed by a coked injector nozzle (naturally coked obtained from the engine department of the repair shipyard). Three out of nine ports are clogged.

Task 2. Measurement of pressure in cylinders, pressure in the fuel injection system, and torsional vibrations in the diesel–electric unit’s shaft—performed in a marine engine with one coked injector nozzle (Figure 4) moved from one cylinder to the next. The factory-assigned cylinder number system was relied upon, meaning that cylinder one was nearest to the timing system drive. The task was divided into the following stages:

stage 1—coked injector nozzle in cylinder one,
stage 2—coked injector nozzle in cylinder two,
stage 3—coked injector nozzle in cylinder three.

Task 3. Developing the spectra of the shaft’s torsional vibrations and analyzing these.

The ship’s diesel–electric unit is a generator of vibration signals leading to time-varying shaft torsions. It was assumed that the shaft torsion signal would be the highest at the time the fuel ignites, i.e., once per two crankshaft revolutions. In the case of a three-cylinder engine, this harmonic component should be tripled to obtain the combustion harmonic component of 1$\frac{1}{2}$ (Table 2).

### 3.1. Results of Tests of a Diesel–Electric Power Unit with a Defect-Free Ship Engine

**Figure 5:**
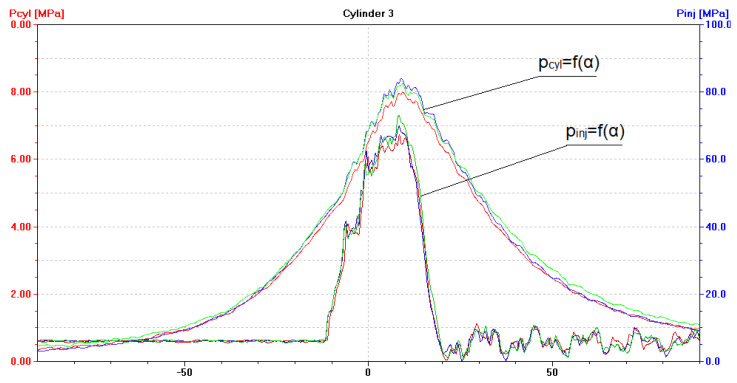
The diagram (screenshot of Unitest 2008) illustrates the p_cyl_ = f(α) function with fuel injection characteristics of p_inj_ = f(α) for a diesel–electric unit (DEU) with a defect-free/healthy ship engine loaded at 70% of MCR. Waveforms for cylinder: ― No. 1, ― No. 2, ― No. 3.

As indicated by the diagrams (Figure 5), fuel injection characteristic curves p_inj_ = f(α), as well as variations in instantaneous cylinder pressure p_cyl_ = f(α), reveal similar waveforms and do not differ significantly in terms of their values.

**Figure 6:**
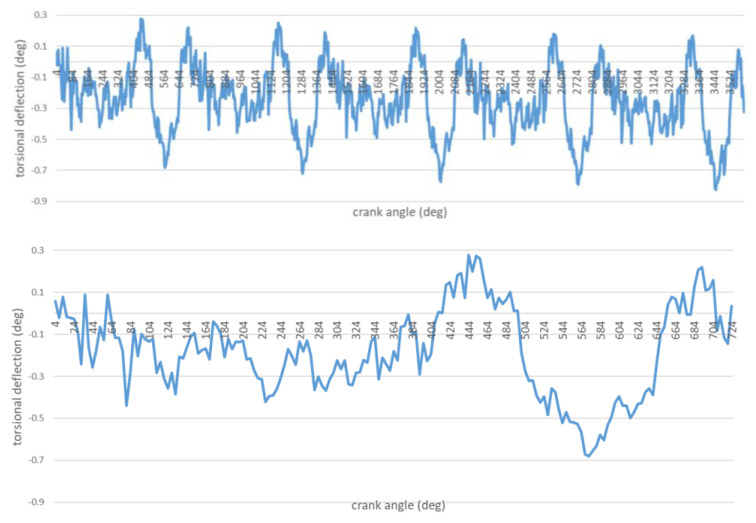
Torsional vibrations of the shaft as a function of the shaft rotation angle. DEU with a defect-free/healthy ship engine (ten turns and one cycle).

The maximum fuel injection pressure values p_inj_ are slightly different, while the angles at which the injection of fuel commences have the same value. This proves that the static adjustment of the fuel injection system was correct. Torsional vibration waveforms in the case of a unit operating under such a load are shown in Figure 6.

**Figure 7:**
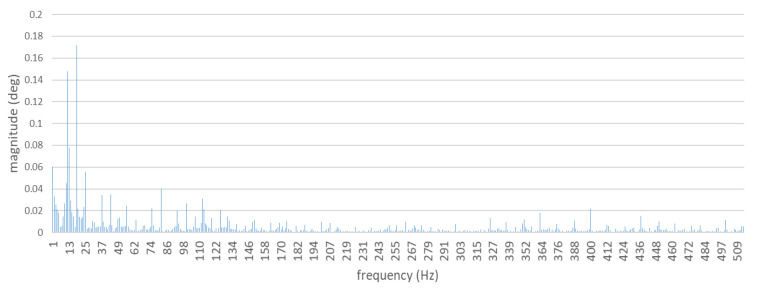
Full spectrum of torsional shaft vibrations. Diesel–electric unit with a defect-free ship engine.

**Figure 8:**
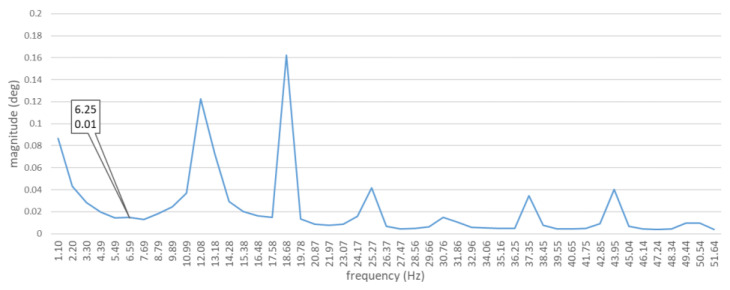
Mean value of torsional shaft vibration spectra. The diagram shows the 8th harmonic component only. Diesel–electric power unit with a defect-free ship engine.

In order to generate the spectra, torsional vibrations recorded were subjected to the discrete Fourier transform (DFT). The spectra obtained are shown in Figure 7 and Figure 8.

For the total of 124 measurements of the shaft torsional vibrations and the prepared amplitude-frequency spectra, the matrices were developed, and the average Pearson’s correlation coefficients were calculated. Comparisons were made for full spectra, and also truncated to the first sixteen harmonics. The obtained results are characterized by a strong correlation (Table 3).

For the speed of the tested engine equaling 750 rpm, the value of the combustion harmonic component k = 1$\frac{1}{2}$ was 18.75 Hz, while the frequency of the basic harmonic component was 12.5 Hz. (Table 2). After decomposition of the signal, these two main harmonic components are clearly visible in the spectrum (Figure 8). The diagram also shows other harmonic components of orders 2 (25 Hz), 2$\frac{1}{2}$ (31.25 Hz), 3 (37.5 Hz), and 3$\frac{1}{2}\;($43.5 Hz). In accordance with the previous assumption that was supported by the literature [20], it was the harmonic component of combustion which achieved the highest amplitude value.

### 3.2. Results of Tests with a Diesel–Electric Power Unit and a Coked Injector Nozzle

**Figure 9:**
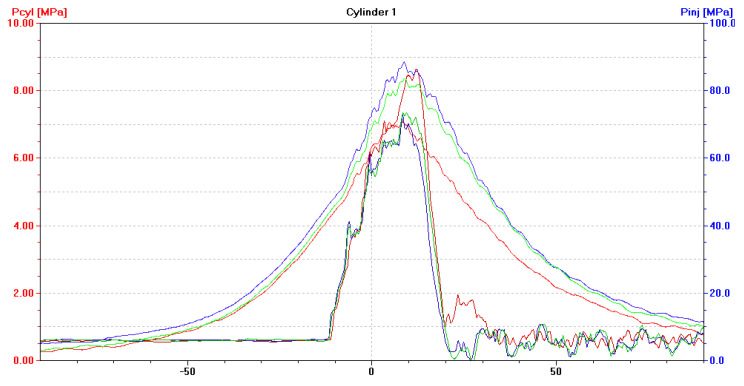
The diagram (screenshot of Unitest 2008) illustrates p_cyl_ = f(α) with the fuel injection characteristics of p_inj_ = f(α) for a DEU with a defective ship engine-coked injector nozzle in the first cylinder, loaded at 70% MCR. Waveforms for cylinder: ― No. 1, ― No. 2, ― No. 3.

**Figure 10:**
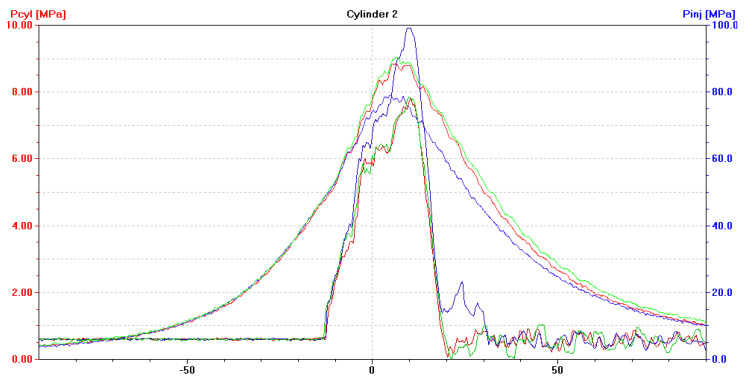
The diagram (screenshot of Unitest 2008) illustrates p_cyl_ = f(α) with the fuel injection characteristics of p_inj_ = f(α) for DEU with a defective ship engine-coked injector nozzle in the second cylinder, loaded at 70% MCR. Waveforms for cylinder: ― No. 1, ― No. 2, ― No. 3.

**Figure 11:**
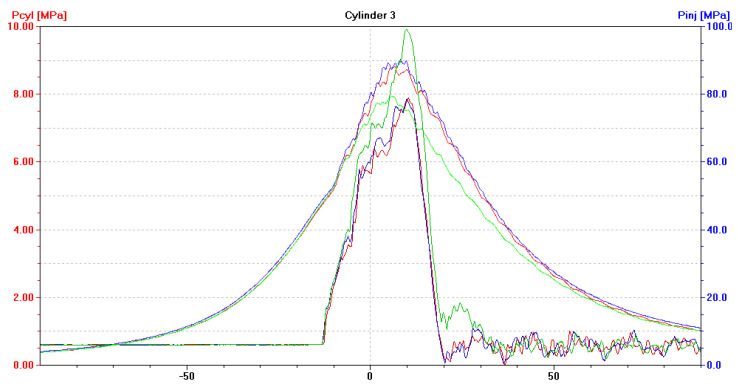
The diagram (screenshot of Unitest 2008) illustrates p_cyl_ = f(α) with the fuel injection characteristics of p_inj_ = f(α) for DEU with a defective ship engine-coked injector nozzle in the third cylinder, loaded at 70% MCR. Waveforms for cylinder: ― No. 1, ― No. 2, ― No. 3.

After installing a coked injector nozzle (in one cylinder at a time), the following have been recorded:

(a) An increase in the pressure of fuel injected by the coked nozzle, to approximately 100 MPa (Figure 9, Figure 10 and Figure 11). Injectors in a defect-free engine spray fuel at the pressure of approximately 70 MPa (Figure 5). The difference was 30 MPa and maximum permissible error in this case was 0.8 MPa;
(b) An increase in the pressure of fuel sprayed by the two remaining, non-defective injectors, to approximately 75 MPa (Figure 9, Figure 10 and Figure 11);
(c) A drop of the maximum combustion pressure in the cylinder with the coked injector, to approximately 7 MPa (Figure 9, Figure 10 and Figure 11). The maximum combustion pressures in cylinders of a defect-free engine were almost equal and amounted to approximately 8 MPa (Figure 5). The difference was 1 MPa and the maximum permissible error in this case was 0.075 MPa;
(d) An increase in the maximum combustion pressure in the two remaining, defect-free cylinders, up to approximately 9 MPa (Figure 9, Figure 10 and Figure 11).

**Figure 12:**
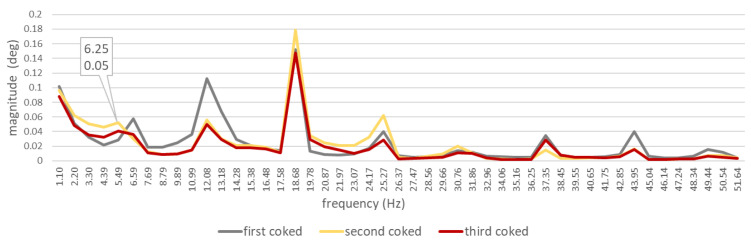
Mean value of torsional shaft vibration spectra. The diagram is limited to the 8th harmonic component only. Ship engine with a coked injector nozzle.

This means that the differences in torque values generated by each cylinder will be greater and, consequently, the pulsation/distribution of torsional forces affecting the drive shaft will be changed. In this case, the recorded waveforms of torsional vibrations were also subjected to the discrete Fourier transform (DFT) in order to obtain the spectra. The spectra obtained are shown in Figure 12. The spectra of torsional vibrations of a unit with a coked injector nozzle, installed in one cylinder at a time, show a clear domination of the combustion harmonic component’s amplitude. After a preliminary analysis, it was found that, unlike in the case of spectra obtained for a defect-free ship engine, a harmonic component of the order of $\frac{1}{2}$ (6.25 Hz) was clearly visible. This component corresponds to the combustion of a fuel–air mixture in a single cylinder (Table 2).

**Figure 13:**
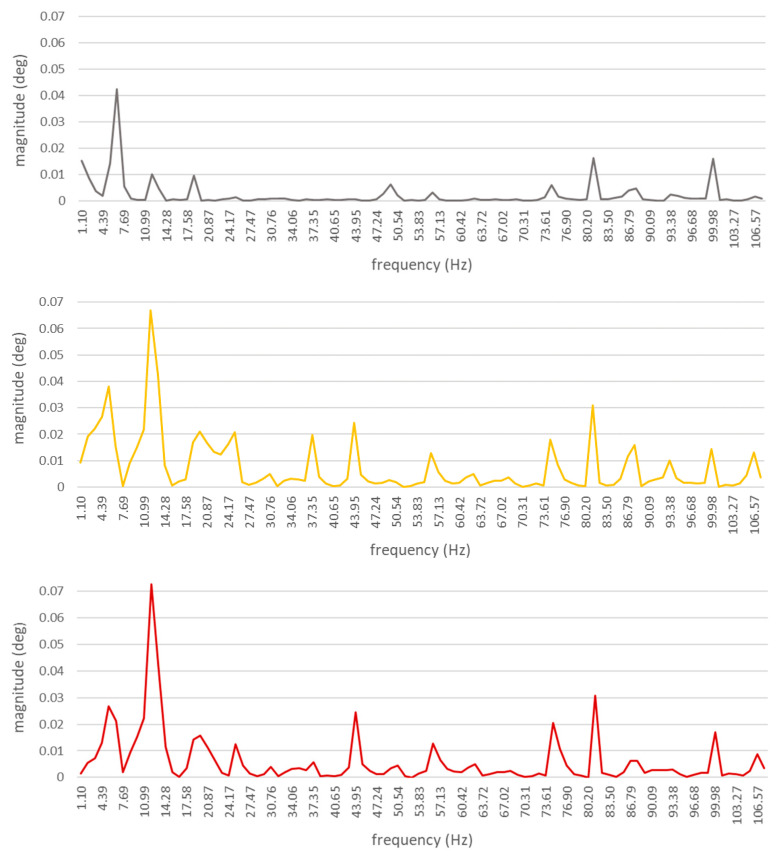
Absolute values of differences in the spectra related to a diesel–electric unit with a defect-free engine and with a defective injector nozzle. The diagram is limited to the 16th harmonic component only. Waveforms for: differences between defect-free and first coked; differences between defect-free and second coked; differences between defect-free and third coked.

Torsional vibration spectra of the DEU with a defective injector nozzle were different from those obtained for a defect-free engine (Figure 13):

― With the first injector nozzle defective, the largest differences in amplitude values were observed for seven harmonic components of the following orders: $\frac{1}{2}$ (6.25 Hz), 1 (12.5 Hz), $1\frac{1}{2}$ (18.75 Hz), 4 (50 Hz), 6 (75 Hz), 6$\frac{1}{2}$ (81,25 Hz), and 8 (100 Hz);
― With the second injector nozzle defective, the largest differences in amplitude values were observed for twelve harmonic components of the following orders: $\frac{1}{2}$ (6.25 Hz), 1 (12.5 Hz), $1\frac{1}{2}$ (18.75 Hz), 2 (25 Hz), 3 (37.5 Hz), $3\frac{1}{2}$ (43.5 Hz), $4\frac{1}{2}$ (56.25 Hz), 6 (75 Hz), 6$\frac{1}{2}$ (81,25 Hz), 7 (87.5 Hz), 7$\frac{1}{2}$ (93.75), and 8 (100 Hz);
― With the third injector nozzle defective, the largest differences in amplitude values were observed for nine harmonic components of the following orders: $\frac{1}{2}$ (6.25 Hz), 1 (12.5 Hz), $1\frac{1}{2}$ (18.75 Hz), 2 (25 Hz), 3$\frac{1}{2}$ (43.5 Hz), 4$\frac{1}{2}$ (56.25 Hz), 6 (75 Hz), 6$\frac{1}{2}$ (81,25 Hz), 8 (100 Hz).

## 4. Conclusions

The results obtained while testing the system for measuring torsional vibrations, designed and built at the Maritime University of Gdynia, allow us to conclude that:

(a) The assumptions adopted for the proprietary algorithm used for calculating torsional vibration values were correct;
(b) The data, recorded by 16 MHz laser heads, are sufficient to determine torsional vibrations of the diesel–electric unit’s shaft;
(c) The spectra obtained for the defect-free/healthy ship engine are strongly correlated (Table 3). It proves the high repeatability of the results for a given sample;
(d) The spectra obtained for the engine with a particular coked injector are strongly correlated (Table 3), which also proves the high repeatability of the results for a given sample;
(e) The values of harmonic component orders obtained are clearly visible in the spectra (Figure 8);
(f) Torsional vibration spectra of the DEU with a defective injector nozzle were different from those obtained for a defect-free engine (Figure 13);
(g) The distribution of differences in the values of the first sixteen harmonic components depend on the cylinder in which the defective injector nozzle was installed.

The observations made lead to a conclusion that it is possible to diagnose coking of a ship diesel engine injection nozzle by relying on spectral analysis of the shaft’s torsional vibrations which are measured by optical sensors.

## Figures and Tables

**Table 1:** Test-stand technical and nominal parameters.

**Sulzer 3AL25/30 Four-Stroke Engine**		
Piston diameter	250	(mm)
Piston stroke	300	(mm)
Nominal effective power	408	(kW)
Mean effective pressure	1.47	(MPa)
Injector opening pressure	25	(MPa)
Fuel delivery advance angle	17	(deg)
Nominal rotating speed	750	(rpm)
Number of cylinders	3	(-)
Firing order	3-2-1	(-)
**GD8 500-50/3 Synchronous Generator**		
Power	500	(kVA)
Rotating speed	750	(rpm)
Stator voltage	400	(V)
Stator current	723	(A)
Frequency	50	(Hz)

**Table 2:** Orders of selected harmonic components and corresponding frequencies.

Order of a Harmonic (k)	Frequency (Hz)
$\frac{1}{2}$	6.25 (one cylinder combustion)
1	12.5 (basic harmonic component)
1$\frac{1}{2}$	18.75 (combustion harmonic component)
2	25
2$\frac{1}{2}$	31.25
3	37.5
3$\frac{1}{2}$	43.5
4	50 (polar pulsation for four pairs of poles of a single voltage phase)

**Table 3:** Sample Pearson’s correlation coefficient.

Engine Condition	Sample Pearson’s Correlation Coefficient Full Spectra/First Sixteen Harmonics
Defect-free/healthy engine	0.84/0.83
Coked injector nozzle in the first cylinder	0.80/0.78
Coked injector nozzle in the second cylinder	0.92/0.93
Coked injector nozzle in the third cylinder	0.82/0.81

## Data Availability

Not applicable.
